# Developmental consequences of children born from mothers with telbivudine treatment during late pregnancy: A prospective study with 3-year follow-up

**DOI:** 10.1080/21505594.2021.1936769

**Published:** 2021-06-12

**Authors:** Wei Yi, Xiuzhen Cao, Zhan Zeng, Weihua Cao, Ying Zhang, Fangfang Sun, Ying Wang, Gang Wan, Minghui Li, Yao Xie

**Affiliations:** a Department of Gynecology and Obstetrics, Beijing Ditan Hospital, Capital Medical University, Beijing, China; b Department of Hepatology Division 2, Peking University Ditan Teaching Hospital, Beijing, China; c Department of Hepatology Division 2, Beijing Ditan Hospital, Capital Medical University, Beijing, China; d Department of Child Health Care, Beijing Children’s Hospital, Capital Medical University, Beijing, China; e Medical Records and Statistics Room, Beijing Ditan Hospital, Capital Medical University, Beijing, China

**Keywords:** Neurological development, chronic hepatitis B, pregnancy, safety, telbivudine

## Abstract

We prospectively investigated the neurological development in infants born from mothers treated with telbivudine (LdT) in the third trimester for prevention of hepatitis B virus (HBV) mother-to-infant transmission. Mothers with high HBV load were assigned to either the LdT group (*n* = 81, 600 mg of LdT each day from gestational week 28 to delivery) or the Control group (*n* = 39, untreated). Their infants were followed for 36 months to assess physical and neurological developments with Gesell Developmental Schedule tools. At 12 months after birth, the mean scores in the LdT group for gross motor, fine motor, adaptive, linguistic, and personal social domains were similar to those in the Control group. At 36 months, infants in the LdT group had higher mean scores for gross motor than the Control group (98.42 ± 9.69 vs. 94.54 ± 7.48, *P* = 0.03). In the LdT group, the rates of normal development were higher for gross motor (96.30% vs. 82.05% *P* = 0.01) and lower for adaptive (74.07% vs. 92.31% *P* = 0.02). Multivariate regression analyses showed that exposure to LdT during pregnancy was independently associated with infant’s development in gross motor (OR 6.49, 95% CI 1.37–30.20, *P* = 0.02) and adaptive (OR 0.18, 95% CI 0.05–0.71, *P* = 0.01) at 36 months. These results suggest that prenatal LdT exposure might affect neurological development in long-term observation.

Abbreviations: LdT: telbivudine; HBV: hepatitis B virus; HBsAg: hepatitis B surface antigen; HBeAg: hepatitis Be antigen; HbsAb: hepatitis B surface antibody; ALT: alanine aminotransferase; NA: nucleoside/nucleotide analog; LAM: lamivudine; MTCT: mother-to-child transmission; GDS: Gesell Developmental Schedule; OR: odds ratio; CI: confidence interval; DQ: developmental quotient; RMB: renminbi; BMI: body mass Index; HBIG: hepatitis B immunoglobulin.

## Introduction

Approximately 2 billion people worldwide are infected with the hepatitis B virus (HBV), and ~292 million people live with chronic infections [1]. An estimated 0.88 million people die of HBV infection-related liver failure, cirrhosis, and primary cancer every year [1]. Mother-to-infant transmission (MTCT) is the most common and important route for chronic HBV infection in Asia [2–4]. Although standard immunoprophylaxis with a birth dose of hepatitis B immunoglobulin (HBIG) and series HBV vaccine has been effective in preventing MTCT among mothers with HBeAg negative, a significantly higher rate of MTCT was observed among HBeAg-positive mothers with a high level of HBV viremia [5–8]. Several studies found that about 8%–30% of infants who were born to mothers with a high level of HBV DNA (above 200,000 IU/mL) become infected with HBV [7,9–11]. Thus, many guidelines have recommended antiviral therapy during pregnancy for CHB infected mothers with high levels of HBV DNA.

Telbivudine (LdT), tenofovir disoproxil, and lamivudine are currently recommended by the American Association for the Study of Liver Diseases (AASLD) for administration in late pregnancy to women with a high HBV DNA load [12]. Several large cohort studies have demonstrated the efficacy and safety of using tenofovir or tenofovir disoproxil during pregnancy for preventing MTCT in this special population [11,13]. Administration of LdT to HBV-infected mothers, started during early, middle, or third trimester of pregnancy, could block mother-to-infant HBV transmission on the basis of standard immune prophylaxis procedure [14–16]. Although the efficacy and short-term safety of LdT on preventing MTCT have been demonstrated, there are little data on the safety assessment of the infant’s long-term neurological development after prenatal exposure to LdT [17]. We know that fetal nervous system development has a key stage at which brain cell differentiation, migration, myeloid, dendritic, and synaptic development, the establishment of neural connections, and glial cell proliferation are completed. It begins during the second trimester and reaches its peak at birth, so it is possible that LdT might affect neurological development even if we start antiviral treatment in the third trimester [18]. Therefore, we conducted a prospective cohort study to continuously investigate the long-term effect of prenatal LdT exposure on infant’s growth and development during a follow-up of 3 years

## Materials and methods

### Population

Pregnant mothers with CHB and treatment naïve who attended prenatal care clinic at Beijing Ditan Hospital from November 2014 to December 2015 were screened at gestational weeks 24–26 and enrolled at gestational week of 28. All cases were of Han nationality from northern China. The study was approved by the Institutional Review Board of Beijing Ditan Hospital (Approval number JDL2014-076-01). Written informed consents were obtained from all participants, and the study was registered at clinicaltrials.gov (Clinical Trials. gov ID: NCT02301650).

Pregnant mothers who met the inclusion and exclusion criteria were enrolled. The inclusion criteria of the study were as follows: age between 20 and 35 years old, with HBsAg and HBeAg positive >6 months, and serum HBV DNA load >10^6^ IU/mL, and willing to provide written informed consent and compliance with the protocol. The exclusion criteria of the study were as follows: (1) history of amniocentesis during pregnancy [19]; (2) history of familial genetic disease in the mother or father; (3) mother received antiviral therapy or HBIG injection therapy during pregnancy; (4) coinfection with hepatitis C virus, hepatitis D virus, human immunodeficiency virus, syphilis, toxoplasmosis, rubella, or cytomegalovirus; (5) history of two or more spontaneous abortions; (6) previous delivery of a child with a deformity; (7) twin or multiple pregnancy; (8) mother had history of cancer; (9) unwilling to complain with follow-up schedule or delivery in our center; and (10) fetal abnormality during pregnancy detected by imagine study or other tests including genetic testing. .

### Study procedure

At the time we recruited patients in 2015, antiviral therapy during pregnancy remained a topic of controversy because WHO guidelines in 2015 recommended against the use of antiviral treatment during pregnancy to prevent MTCT. Therefore, based on the mother’s preference patients were free to choose to receive 600 mg of LdT each day from gestational week 28 to delivery (LdT group) or receive usual care without any antiviral treatment (Control group). At the first visit at gestational week 24–26, the demography and other baseline data, including living habit, education, household financial income, mother’s pregnancy and gestation history, were collected and HBV DNA load, HBsAg and HBeAg levels, liver and kidney function were tested. The pregnancy and delivery complications were recorded at any time.

Infants received standard immunoprophylaxis (HBIG 200 IU, Chengdu Institute of Biological Products, Chengdu, China) and HBV vaccine (10 μg; Dalian Hissen Biopharm Co., China) within 6 h, additional two doses of HBV vaccine given at the age of 1 month and 6 months. Infant’s weight, height and Apgar score at 1 min, congenital malformations, and adverse events at birth were recorded. In subsequent visits from birth to 36 months, the data of adverse events, history of hospitalization, feeding status, parenting patterns, and other vaccinations were recorded. At 12 months and 36 months after birth, HBV serological markers, HBV DNA load, infants’ physical, and neuro-mental developments were assessed.

To avoid the influence of researchers on the results of development measurement and reduce any bias, in this study, the assessments of neurological development were performed by an independent professional from another hospital blind to the information of mothers and children, including the drug used during pregnancy.

### Study endpoint

Primary endpoints were infant’s safety, including congenital defect rates, the neurological and physical development. Infants’ neurological development was evaluated at 12 and 36 months after birth using the Gesell Developmental Schedule (GDS) assessment tools, which has five domains including gross motor, fine motor, linguistic, adaptive, and personal-social skills. Adaptive domain means the ability to adapt to a new environment, to analyze and deal with the external stimuli and to use past experience to solve new problems. Every infant was assigned a developmental age (DA) for each of the five areas. When analyzing the developmental scores, the corrected chronological age (CCA) of each infant was first calculated from the infant’s expected date of birth. The developmental quotient (DQ) was defined as DA/CCA x100. Subsequently, the quantitative comparable index scores corresponding to each tested domain were recorded based on the GDS raw scores. The normal neurological development was defined as DQ ≥ 86%, whereas the developmental delay was defined as DQ ≤ 75%. When the infant’s DQ was at the range of 76%–85%, the delay on the neurological development was suspected. The physical growth was assessed with mean body weight (kg) and height (cm) of each group at 12 and 36 months.

The secondary endpoints include the following: (1) the rates of MTCT defined as infants having HBAg positivity or detectable levels of HBV DNA at the age of 12 months, (2) adverse events and complications of infants from birth to 36 months, and (3) maternal safety, including adverse events and complications during pregnancy.

### Laboratory tests

All laboratory tests were performed at the Central Laboratory of Beijing Ditan Hospital. HBsAg, HBsAb, and HBeAg were detected by a chemiluminescent microparticle immunoassay (Architect i2000 analyzer; Abbott Diagnostics, Abbott Park, IL, USA). The detection range of HBsAg level was 0.05–250 IU/mL, the detection range of HBsAb level was 0–1000 mIU/mL, and the detection limit of HBeAg was 1.0 S/CO (sample/cut off). Serum HBV DNA load was quantified using a real-time fluorescent quantitative PCR amplification (Kehua Biological Company, Shanghai, China), with a lower limit of 500 copies/mL. Blood routines were run on a Sysmex XE-5000 system (Japan), while liver and kidney function was tested using a Hitachi 7600 biochemical analyzer (Hitachi 7600–020, Japan).

The auditory screening was performed using the Madsen Echo Screen (Germering, Germany). After 72 h of breastfeeding or artificial feeding, heel blood was collected on filter paper from the infants and tested by the Beijing Neonatal Diseases Screening Center to rule out congenital phenylketonuria and hypothyroidism. The same physician completed all GDS examinations from the Beijing Child Health Care Institute.

### Statistical analysis

Descriptive statistics were used to analyze the characteristics of the study population. Continuous variables were expressed as means ± standard deviation or quartile division. Student’s *t*-test was applied to analyze continuous variables between two groups. Categorical variables were described using numbers and percentages. The chi-square and Fisher’s exact tests were used to compare rates between two groups. A rank-sum test was used to compare ordinal data. Multivariate regression was used to analyze the independent effect of LdT on neurodevelopmental outcomes. All statistical analysis was performed using SPSS software program, version 17.0 (SPSS Inc., Chicago, IL, USA). A *P*-value of <0.05 was considered statistically significant.

## Results

### Sociodemographic characteristics

**Figure 1.**
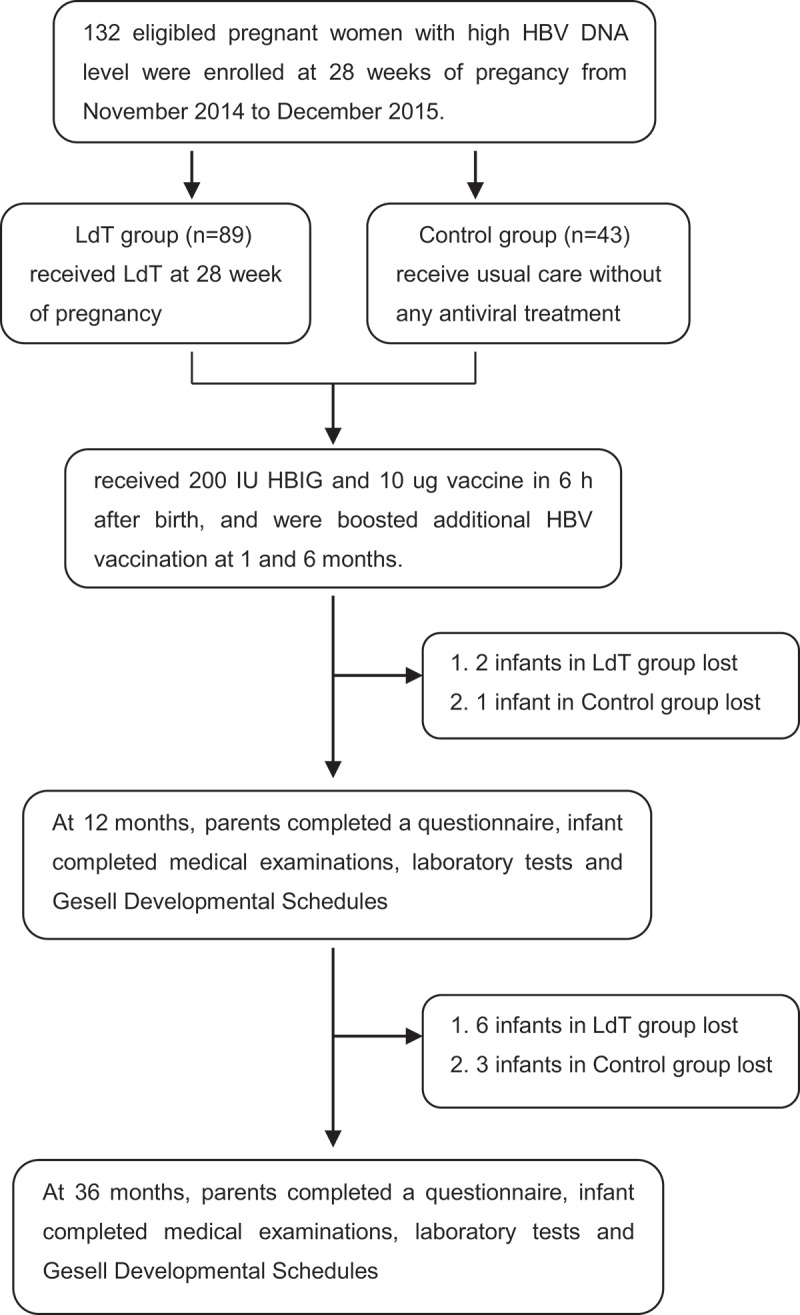
Enrollment of the trial participants

Abbreviations: LdT: telbivudine; HBV: hepatitis B virus; HBIG: hepatitis B immunoglobulin

Among 1482 pregnant women with CHB who attended the prenatal care clinic at Beijing Ditan Hospital from November 2014 to December 2015, 428 mothers had high levels of viremia and were eligible for the study, but 296 patients were unwilling to consent or unable to comply with the follow-up schedule. The remaining 132 mothers who met the inclusion and exclusion criteria were enrolled in the study (89 in LdT group, 43 in Control group). The patients’ deposition is presented in Figure 1. At the end of the study, 81 mother–infant pairs in the treated group and 39 in the Control group completed the 36 months of follow-up. The baseline characteristics were similar between the LdT group and the Control group, except that the younger age and higher maternal levels of HBV DNA were presented in the LdT group (Table 1). The mean duration of LdT treatment before delivery for mothers in the LdT group was 11.8 ± 0.9 weeks.

**Table 1.** Characteristics of the parents at baseline and the infants at delivery

Characteristic of the parents at baseline	LdT group *n* = 81 (%)	Control group *n* = 39 (%)	*Z* or *t* or *x^2^*	*P*
**Maternal character**				
**Maternal age (years**), mean±SD	26.63 ± 3.27	28.18 ± 3.47	*2.38*	0.02*
**Gravidity (times)**	1.48 ± 0.76	1.49 ± 0.82	*0.04*	*0.97*
**Parity (times)**	1.05 ± 0.21	1.21 ± 0.46	*1.97*	*0.054*
Maternal education time (years), ***n*** (%)				
≤9	9(11.11)	7(17.95)	*−1.42*	*0.16*
10–12	22(27.16)	3(7.69)		
13–16	44(54.32)	22(56.41)		
17–19	6(7.41)	5(12.82)		
≥20	0	2(5.13)		
**Household annual gross income (10,000 RMB yuan)**				
<5	6(7.41)	3(7.69)	*−1.15*	*0.25*
5–10	26(32.10)	8(20.51)		
10–20	29(35.80)	15(38.46)		
20–50	16(19.75)	11(28.21)		
>50	4(4.94)	2(5.13)		
**Prepregnancy BMI (kg/m^2^)**				
<18.5	14(17.28)	6(15.38)	*−0.27*	*0.79*
18.5–23.9	53(65.43)	26(66.67)		
24.0–27.9	12(14.81)	5(12.82)		
≥28.0	2(2.47)	2(5.13)		
Smoking prior to pregnancy, ***n*** (%)	2(2.47)	0	*Fisher*	*1.00*
Alcohol prior to pregnancy, ***n*** (%)	3(3.70)	0	*Fisher*	*1.00*
**HBV DNA load (log_10_ copies/mL)**	7.43 ± 0.51	6.96 ± 1.08	*−2.52*	0.02*
**ALT (U/L**), mean±SD	24.52,22.23	21.06 ± 10.76	*−0.90*	*0.37*
**Paternal character**				
**Paternal age (years**), mean±SD	27.89 ± 3.94	29.18 ± 4.00	*1.67*	*0.10*
Paternal education time (years), ***n*** (%)				
≤9	7(8.64)	5(12.82)	*0.66*	*0.51*
10–12	19(23.46)	4(10.26)		
13–16	47(58.02)	26(66.67)		
17–19	6(7.41)	2(5.13)		
≥20	2(2.47)	2(5.13)		
Prepregnancy BMI (kg/m^**2**^), ***n*** (%)				
<18.5	2(2.47)	1(2.56)	*1.65*	*0.65*
18.5–23.9	34(41.98)	20(51.28)		
24.0–27.9	30(37.04)	14(35.90)		
≥28.0	15(18.52)	4(10.26)		
Smoking prior to pregnancy, ***n*** (%)	33(40.74)	13(33.33)	*0.04*	*0.84*
Alcohol prior to pregnancy, ***n*** (%)	10(12.35)	8(20.51)	*0.61*	*0.43*
Characteristic of the infants at delivery	LdT group n = 81 (%)	Control group n = 39 (%)	Z or t or x^2^	*P*
**Gestation time (day)**	278.60 ± 5.74	275.72 ± 7.53	*−2.32*	0.02*
**Male (%)**	43(53.09)	20(51.28)	*0.03*	*0.95*
**Cesarean** **section (%)**	27(33.33)	21(53.85)	*4.62*	0.03*
**Neonatal weight (g)**	3424.69 ± 373.50	3394.87 ± 382.64	*−0.41*	*0.69*
**Neonatal height (cm)**	50.10 ± 0.40	50.18 ± 0.79	*0.74*	*0.46*
**1 min Apgar score**	9.95 ± 0.31	10.00 ± 0.00	*0.99*	*0.33*
**Malformation (%)**	3 (3.70)	0	*Fisher*	*1.00*
**Parenting pattern**				
Parents	13(16.05)	2(5.13)		
Eldership	2(2.47)	0		
Housekeeper	0	0	*4.03*	*0.13*
Relative	0	0		
Dual culture	66(81.48)	37(94.87)		
**Feeding status**				
Breast feeding	3(3.70)	3(7.69)		
Artificial feeding	74(91.36)	28(71.79)	*8.41*	*0.02*
Mixed feeding	4(4.94)	8(20.51)		

*Had significant difference

Ltd: telbivudine; RMB: renminbi; BMI: body mass Index; HBV: hepatitis B virus; ALT: alanine aminotransferase

### Primary outcome

#### Infants’ congenital defect rates and physical development

Three infants in the LdT group had congenital malformations, including one accessory ear, one congenital pyloric stenosis (treated with surgical modification at the age of 28 days), and one congenital torticollis (treated surgically at 1 year old). However, the rates of congenital malformations did not differ from that of the Control group (3.70% vs. 0%, *P* = 1.00). In the current study, no infant had congenital phenylketonuria, hypothyroidism, or abnormal results from auditory screening.

At birth, the weight and height of infants in the two groups were similar. After birth, more parents selected artificial feeding in LdT group (91.36% vs 71.79%, *P* < 0.05, Table 1).

At 12 months and 36 months after birth, the infant’s weight, height, BMI, and the rates of infants, who had weight, height or BMI, were comparable between two groups (*P* > 0.05, Table 2).

**Table 2.** Development and GDS scores of infants at 1 year old and 3 years old

**Development varies**	LdT group *n* = 81 (%)	Control group *n* = 39 (%)	*Z* or t or *x*^2^	*P*
**At 1-year old** **Average age (day)**	397.99 ± 16.98	397.38 ± 18.52	*−0.18*	*0.86*
**Height (cm)**				
Average	77.62 ± 2.75	77.66 ± 3.17	*0.08*	*0.94*
Average, boys	78.23 ± 2.83	78.40 ± 2.64	*0.22*	*0.83*
Average, girls	76.92 ± 2.52	76.89 ± 3.55	*−0.04*	*0.97*
Higher than reference range (%)	14(17.28)	6(15.38)	*0.07*	*0.79*
Lower than reference range (%)	2(2.46)	0(0.00)	*Fisher*	*1.00*
**Weight (kg)**				
Average	10.66 ± 1.14	10.60 ± 1.13	*−0.31*	*0.76*
Average, boys	10.97 ± 1.11	11.03 ± 1.03	*0.21*	*0.83*
Average, girls	10.32 ± 1.10	10.14 ± 1.07	*−0.60*	*0.55*
Higher than reference range (%)	11(13.58)	3(7.69)	*Fisher*	*0.55*
Lower than reference range (%)	0(0.00)	0(0.00)	*Fisher*	*1.00*
**BMI (kg/m^2^)**				
Average	17.71 ± 1.76	17.56 ± 1.53	*−0.44*	*0.64*
Average, boys	17.95 ± 1.90	17.95 ± 1.54	*0.01*	*1.00*
Average, girls	17.44 ± 1.56	17.15 ± 1.44	*−0.68*	*0.50*
Higher than reference range (%)	10(12.3)	3(7.70)	*Fisher*	*0.54*
Lower than reference range (%)	0(0.00)	0(0.0)	*Fisher*	*1.00*
**At 3-year old** Average age (day)	1063.42 ± 182.31	1086.41 ± 156.38	*0.68*	*0.50*
**Height (cm)**				
Average	97.82 ± 4.47	98.47 ± 3.93	*0.68*	*0.50*
Average, boys	97.99 ± 4.80	99.71 ± 4.03	*1.27*	*0.21*
Average, girls	97.60 ± 4.05	96.96 ± 3.36	*−0.49*	*0.62*
Higher than reference range (%)	2(2.46)	0(0.00)	*Fisher*	*1.00*
Lower than reference range (%)	0(0.00)	0(0.00)	*Fisher*	*1.00*
**Weight (kg)**				
Average	15.02 ± 1.81	15.25 ± 1.77	*0.60*	*0.55*
Average, boys	15.08 ± 2.12	15.72 ± 2.00	*1.04*	*0.31*
Average, girls	14.93 ± 1.30	14.69 ± 1.32	*−0.55*	*0.59*
Higher than reference range (%)	2(2.46)	0(0.00)	*Fisher*	*1.00*
Lower than reference range (%)	0(0.00)	0(0.00)	*Fisher*	*1.00*
**BMI (kg/m^2^)**				
Average	15.71 ± 1.79	15.69 ± 1.15	*−0.05*	*0.96*
Average, boys	15.73 ± 2.16	15.76 ± 1.40	*0.06*	*0.96*
Average, girls	15.68 ± 1.11	15.60 ± 0.80	*−0.23*	*0.82*
Higher than reference range (%)	2(2.47)	0(0.00)	*Fisher*	*1.00*
Lower than reference range (%)	5(6.17)	0(0.00)	*Fisher*	*0.17*
**GDS test parameter**				
	LdT group *n* = 81	Control group *n* = 39	*t*	*p*
**At 1-year old** Gross motor Fine motor Adaptive Linguistic Personal social	104.04 ± 13.68 96.69 ± 8.12 97.27 ± 6.64 87.27 ± 9.44 113.56 ± 9.42	101.36 ± 9.18 94.97 ± 6.92 95.08 ± 6.31 87.05 ± 9.36 111.87 ± 8.04	*−1.27* *–1.14* *-1.72* *–0.12* *-0.96*	*0.21* *0.26* *0.09* *0.91* *0.34*
**At 3-year old** Gross motor Fine motor Adaptive Linguistic Personal social	98.42 ± 9.69 97.11 ± 6.81 90.37 ± 6.19 96.58 ± 9.55 102.05 ± 10.88	94.54 ± 7.48 98.56 ± 5.68 91.36 ± 4.15 95.54 ± 9.51 101.49 ± 7.09	*−2.20* *1.15* *1.03* *–0.56* *-0.29*	0.03* 0.25 *0.30* *0.58* *0.77*

Ltd: telbivudine; BMI: body mass index. GDS: Gesell Developmental Schedule

*Had significant difference

#### Infants’ neurological developmental

At 12 months, there was no statistical difference between the two groups in prespecified neurological development outcomes including GDS scores for gross motor (104.04 ± 13.68 vs. 101.36 ± 9.18, *P* = 0.21), fine motor (96.69 ± 8.12 vs. 94.97 ± 6.92, *P* = 0.26), adaptive (97.27 ± 6.64 vs. 95.08 ± 6.31, *P* = 0.09), linguistic (87.27 ± 9.44 vs. 87.05 ± 9.36, *P* = 0.91), and personal social (113.56 ± 9.42 vs. 111.87 ± 8.04, *P* = 0.34) domains. In both groups, linguistic ability scores were lower than other scores, but no statistically significant difference was observed (Table 2). The percentages of delay, suspicion, and normal development in both groups in the five domains were similar (Table 3).

**Table 3.** The developmental state in the five domains

GDS test parameter	DQ	1-year old				3-year old			
		LdT group *n* = 81	Control group *n* = 39	*z*	*p*	LdT group n = 81	Control group n = 39	*z*	*p*
Gross motor	DQ ≤ 75%	0(0.00)	0(0.00)	*Fisher*	***0.43***	0(0.00)	0(0.00)	*Fisher*	0.01*
	76% ≤ DQ ≤ 85%	6(7.41)	1(2.56)			3(3.70)	7(17.95)		
	DQ ≥ 86%	75(92.59)	38(97.44)			78(96.3)	32(82.05)		
Fine motor	DQ ≤ 75%	0(0.00)	0(0.00)	*Fisher*	*1.00*	0(0.00)	0(0.00)	*Fisher*	*1.00*
	76% ≤ DQ ≤ 85%	4(4.94)	1(2.56)			2(2.47)	0(0.00)		
	DQ ≥ 86%	77(95.06)	38(97.44)			79(97.53)	39(100)		
Adaptive	DQ ≤ 75%	0(0.00)	0(0.00)	*Fisher*	*0.68*	0(0.00)	0(0.00)	*5.470*	0.02*
	76% ≤ DQ ≤ 85%	4(4.94)	3(7.69)			21(25.93)	3(7.69)		
	DQ ≥ 86%	77(95.06)	36(92.31)			60(74.07)	36(92.31)		
Linguistic	DQ ≤ 75%	5(6.17)	3(7.69)	*Fisher*	*0.88*	1(1.23)	1(2.56)	*Fisher*	*0.83*
	76% ≤ DQ ≤ 85%	35(43.21)	18(46.15)			7(8.64)	4(10.26)		
	DQ ≥ 86%	41(50.62)	18(46.15)			73(90.12)	34(87.18)		
Personal social	DQ ≤ 75%	0(0.00)	0(0.00)	*Fisher*	*0.49*	0(0.00)	0(0.00)	*Fisher*	*1.00*
	76% ≤ DQ ≤ 85%	1(1.23)	0(0.00)			2(2.47)	0(0.00)		
	DQ ≥ 86%	80(100)	39(100)			79(97.53)	39(100)		

*Had significant difference

At 3 year old, the score of gross motor in LdT treated group was significant higher than that in Control group (98.42 ± 9.69 vs. 94.54 ± 7.48, *P* = 0.03), but not the GDS scores for fine motor (97.11 ± 6.81 vs. 98.56 ± 5.68, *P* = 0.25), adaptive (90.37 ± 6.19 vs. 91.36 ± 4.15, *P* = 0.30), linguistic (96.58 ± 9.55 vs. 95.54 ± 9.51, *P* = 0.58), or personal social (102.05 ± 10.88 vs. 101.49 ± 7.09, *P* = 0.77) domains (Table 2). In gross motor, the percentage of suspicion was higher in the Control group than that in the LdT group (17.95% vs. 3.7%, *P* = 0.01). In adaptive, the percentage of suspicion was higher in the LdT group than in the Control group (25.93% vs. 7.69%, *P* = 0.02). The percentages of delay, suspicion, and normal development in the other three domains were comparable between the two groups (Table 3).

The multivariate regression by including vitiates of parental education and life habit, household annual gross income, delivery mode, parenting pattern, feeding status, days of gestation, infants’ weight and height at delivery, the days after birth at the investigation time, and LdT exposure was conducted. Results indicated that the DQ in gross motor domain and adaptive domain was independently associated with exposure to LdT during pregnancy (Table 4).

**Table 4.** Association between prenatal exposure to LdT and the neurological developmental scores at 12 months and 36 months after birth (*n* = 130)

GDS	12 m		36 m	
	OR (95%CI)*	P value	OR (95%CI)*	*P-*value
Gross motor	0.26 (0.02–3.14)	*0.29*	6.494(1.40–30.20)	0.02*
Fine motor	0.33(0.02–4.35)	*0.40*	0.00(0.00-)	*0.98*
Adaptive	1.88 (0.25–14.22)	*0.54*	0.18(0.05–0.71)	0.01*
Linguistic	2.49(0.32–19.56)	*0.39*	0.99(0.19–5.18)	*0.99*
Personal social	0.00(0.00-)	*0.99*	0.00(0.00-)	*0.99*

The model included parental education and life habit, household annual gross income, delivery mode, parenting pattern, feeding status, days of gestation, infant's weight and height at delivery, the days after birth at the investigation time, and LdT exposure.

*Had significant difference

Ltd: telbivudine, GDS: Gesell Developmental Schedule; OR: odds ratio; CI: confidence interval.

### Secondary outcome assessment

#### Assessment of maternal and fetal adverse events

The most common adverse event in mothers during pregnancy was diabetes mellitus. The frequency of gestational diabetes in the LdT (34.56%) did not differ from that in the Control groups (41.02%, *P* = 0.49). Other adverse events, such as premature rupture of membranes, postpartum hemorrhage, hypothyroidism, meconium staining of the amniotic fluid, oligohydramnios, and elevated bile acid, were similar in two groups (Table 5). During LdT treatment, one mother had mild (Grade I) elevation of creatine kinase with a normal electrocardiogram.

**Table 5.** Adverse events and laboratory abnormalities during pregnancy and perinatal period

Varies	LdT group *n* = 81 (%)	Control group *n* = 39 (%)	*x*^2^	*P*
Diabetes mellitus* *n*(%)	28(34.56)	16(41.02)	*0.47*	*0.49*
Premature rupture of membranes, *n*(%)	10(12.34)	6(15.38)	*0.21*	*0.65*
Postpartum hemorrhage, *n*(%)	6(7.40)	1(2.56)	*Fisher*	*0.43*
Hypothyroidism, n(%)	6(7.40)	2(5.12)	*Fisher*	*1.00*
Meconium staining of the amniotic fluid^a^, *n*(%)	6(7.40)	2(5.12)	*Fisher*	*1.00*
Oligohydramnios, *n*(%)	2(2.46)	0(0.00)	*Fisher*	*1.00*
ICP, *n*(%)	1(1.23)	2(5.12)	*Fisher*	*0.29*
Fever (%)	63(77.78)	32(82.05)	*0.29*	*0.59*
Diarrhea (%)	56(69.14)	29(74.36)	*0.35*	*0.56*
Exanthema subitum (%)	37(45.68)	18(46.15)	*0.00*	*0.96*
Eczema (%)	27(33.33)	14(35.9)	*0.08*	*0.78*
Pneumonia (%)	8(9.88)	5(12.82)	*0.24*	*0.63*
Nasopharyngitis (%)	2(2.47)	0(0.00)	*Fisher*	*1.00*
Bronchitis (%)	4(4.94)	4(10.26)	*Fisher*	*0.44*
Hand, foot and mouth disease (%)	7(8.64)	2(5.13)	*Fisher*	*0.72*
Hospitalization (%)	11(13.58)	5(12.82)	*0.01*	*0.91*

*Includes gestational diabetes mellitus and pregestational diabetes mellitus.

Ltd: telbivudine

#### Vertical transmission rates and infants’ serological status

The rates of effective blocking HBV MTCT, defined as HBsAg-negativity with undetectable HBV DNA levels at 12 months after birth, were 100% in LdT group and 97.44% in Control group without any significant difference (*P* = 0.33). The rates of infants who had protective levels of HBsAb (>10 mIU/mL) were similar between two groups [96.30% (78/81) and 100% (39/39), *P* = 1.00]. The HBsAb levels in LdT-treated group and Control group were 184.92 (73.17, 466.25) mIU/mL and 247.27 (79.22, 562.22) mIU/mL, respectively (*P* = 0.39). At 3 years after birth, there were 82.72% infants who had protective HBsAb levels with median 75.84 (34.24, 167.54) mIU/mL in LdT group, and 82.05% infants had protective HBsAb levels with median 91.57 (30.63, 224.70) mIU/mL in Control group (*P* = 0.78, Table 6).

**Table 6.** Clinical laboratory examination findings at the 1- and 3-year follow-ups

	**1-year old**				**3-year old**			
	LdT group *n* = 81 (%)	Control group *n* = 39	*t* or x2	*P*-value	LdT group n = 81 (%)	Control group n = 39	t or x2	P value
Alanine aminotransferase (U/L)	21.37 ± 6.40	18.98 ± 4.99	*−2.03*	*0.045*	14.36 ± 4.49	13.79 ± 7.09	*−0.51*	*0.61*
Aspartate aminotransferase (U/L)	39.28 ± 8.14	37.93 ± 6.54	*−0.89*	*0.37*	31.02 ± 5.32	30.24 ± 5.11	*−0.74*	*0.46*
Total bilirubin (umol/L)	5.45 ± 2.61	5.74 ± 1.94	*0.59*	*0.56*	6.94 ± 3.16	7.41 ± 3.24	*0.74*	*0.46*
Albumin(g/L)	44.81 ± 1.96	45.12 ± 1.96	*0.82*	*0.41*	46.69 ± 2.48	45.48 ± 7.69	*−1.23*	*0.22*
Red blood cell (10^9^/L)	4.42 ± 0.27	4.34 ± 0.33	*−1.39*	*0.17*	4.68 ± 0.29	4.64 ± 0.38	*−0.54*	*0.59*
Blood platelet (10^9^/L)	291.03 ± 78.83	293.01 ± 168.95	*0.13*	*0.89*	293.81 ± 72.99	292.12 ± 83.31	*−0.10*	*0.92*
Hemoglobin (g/L)	117.3 ± 06.97	115.51 ± 6.44	*−1.35*	*0.18*	125.12 ± 17.35	125.64 ± 8.09	*0.16*	*0.87*
Blood urea nitrogen (mmol/L)	4.53 ± 0.89	4.21 ± 1.18	*−1.64*	*0.11*	4.25 ± 1.06	4.30 ± 0.76	*0.26*	*0.80*
Blood creatinine (umol/L)	25.58 ± 17.85	21.69 ± 2.61	*−1.35*	*0.18*	28.37 ± 6.02	27.45 ± 5.04	*−0.74*	*0.46*
Blood uric acid (umol/L)	223.91 ± 61.88	232.06 ± 65.49	*0.66*	*0.51*	260.25 ± 66.59	245.71 ± 85.67	*−0.91*	*0.37*
Calcium (mmol/L)	2.52 ± 0.09	2.47 ± 0.27	*−1.41*	*0.16*	2.44 ± 0.09	2.44 ± 0.07	*−0.21*	*0.84*
HBV DNA decetable (%)	0(0.00)	1(2.56)	*Fisher*	*0.33*	0(0.00)	1(2.56)	*Fisher*	*0.33*
HBsAg positive (%)	0(0.00)	1(2.56)	*Fisher*	*0.33*	0(0.00)	1(2.56)	*Fisher*	*0.33*
HBsAb < 10 mIU/mL (%)*	3(3.70)	0	*Fisher*	*1.00*	14(17.28)	7(17.95)	*0.01*	*0.93*
HBsAb≥10 mIU/mL (%)*	78(96.30)	39(100)			67(82.72)	32(82.05)		
HBsAb mIU/mL*	184.92(73.17, 466.25)	247.27(79.22, 562.22)	*0.87*	*0.39*	75.84(34.24,167.54)	91.57(30.63,224.70)	*0.29*	*0.78*

*Except for 1 HBV-infected infants

Ltd: telbivudine; HBsAg: hepatitis B surface antigen. HBsAb: hepatitis B surface antibody.

#### Infants’ adverse events

During the 3-year observation period, the most common adverse events in infants were fever, diarrhea, exanthema subitum, and eczema. There was no difference in the rates of all adverse events between the two groups (Table 5). The results of hematological and biochemistry testing showed that prenatal LdT exposure did not affect liver and kidney function in 1 and 3 years (Table 6).

## Discussion

LdT does not affect human nucleotide/DNA synthesis or adversely affect fetal development [20]. Toxicological research also demonstrated that LdT has no carcinogenic, teratogenic, mutagenic effects, or mitochondrial toxicity [21]. Although LdT is a pregnancy category B medication classified by FDA, the safety data on human fetal exposure are very limited. In the antiretroviral pregnancy registry (APR) database, there are only 245 and 13 mothers who received LdT therapy in the first trimester and the second/third trimesters. Moreover, the birth defects rate does not necessarily reflect the physical development in the long term.

More importantly, infants are very sensitive to neurotoxic effects during the perinatal period [18,22], particularly in the third trimester. It is not clear whether LdT treatment in late pregnancy could affect the development of the fetal nervous system. There have been few studies on the developmental consequences of prenatal LdT exposure during pregnancy. In this study, we investigated the correlation of LdT use in late pregnancy to the infants’ neurological developmental after birth. The results verified that prenatal LdT exposure was effective in preventing MTCT and did not affect physical development in offspring.

At 12 months, the mean scores and development of classification in five domains were not different between LdT group and Control group. However, at 36 months, LdT group had a significantly higher score in gross motor domain than Control group, and a higher rate of normal development in gross motor domain. Unlike the result in gross motor domain, although the mean scores of adaptive domain were not different between LdT treated group and Control group, LdT treated group had a significantly lower rate of normal development in adaptive domain. Result of multivariate regression also indicated that exposure to LdT during pregnancy was significantly associated with the DQ in gross motor domain and adaptive domain at 3 years after birth. In this study, infants exposed to Ldt in pregnancy had higher DQ in the gross motor domain than the Control group at 3 years. This result is not consistent with our original assumption. Ldt is capable of inhibiting replication of mitochondrial DNA; thus, the elevation of creatine kinase can be found in patients with chronic hepatitis B treatment with LdT. It is believed that the use of LdT in pregnancy has a negative effect on the fetus. Our contradictory findings might be due to the small sample size. The actual clinical impact of this difference on infants is unclear, which shall be confirmed in future studies by expanding the sample size.

Health organizations recommend that the GDS be used to assess development, and it is widely used and validated in Asian populations [23–26]. In our study, we applied all aspects of the GDS test to assess the development of the nervous system in infants. Although previous studies suggested that mothers with higher levels of education give birth to infants with higher developmental quotient DQ [27], we did not find significant differences in the education level between the two groups.

Another factor that improves the brain development in infants after birth is breastfeeding. Breastfeed infants demonstrated increased VFM in several brain regions involved in vision, language, and higher-order cognition [28]. Breast milk helps earlier development and maturation of some aspects of the nervous system than formula [29]. In China, although breastfeeding is not opposed, there are no guidelines for recommending breastfeeding in CHB mothers with antiviral treatment before 2019. Women with chronic HBV infections have a lower rate of breastfeeding due to concerns about HBV MTCT or the impact of antiviral drugs on infants. In our study, although there was significant difference of feeding type between LdT treated and untreated groups, the effect of feeding type could not be determined, nor did the result of logistics regression show an association between feeding status and neurodevelopment.

Using antiviral drugs in late pregnancy to reduce MTCT was recommended by guidelines based on many large sample studies, which demonstrated that antiviral therapy can significantly decrease MTCT in mothers with high HBV DNA load. In this study, the LdT treatment in late pregnancy had very little effect in preventing MTCT and lower score in adaptive domain, and the reason may be the small sample size. The risk–benefit ratio of blocking hepatitis B transmission versus producing developmental impairment with the treatment drug should be further investigated by a large sample study.

Several limitations of our study are mainly associated with single-center experience and nonrandomized design. In addition, slightly more patients in the Control group were lost to follow-up when compared to the study group, which may affect the ability to discover small differences in outcome assessment. Lastly, our observation is limited to the age of 36 months. Studies with longer follow-ups will be helpful to establish the long-term safety of LdT therapy during pregnancy.

In conclusion, LdT could lower the perinatal transmission rate in CHB mothers. Prenatal LdT exposure might affect neurological development in long-term observations, which awaits further verification in future by large cohort studies.
